# Impact of Transcatheter Aortic Valve Implantation on Kidney Function

**DOI:** 10.36660/abc.20180356

**Published:** 2019-12

**Authors:** Rita Calça, Rui C. Teles, Patrícia Branco, Augusta Gaspar, João Brito, Tiago Nolasco, Manuel D. Almeida, José P. Neves, Miguel Mendes, Domingos S. Machado, André Weigert

**Affiliations:** 1 Nephrology department, Hospital de Santa Cruz, Centro Hospitalar de Lisboa Ocidental, Lisboa - Portugal; 2 Cardiology department, Hospital de Santa Cruz, Centro Hospitalar de Lisboa Ocidental, Lisboa - Portugal; 3 Cardiotoracic Surgery department Hospital de Santa Cruz, Centro Hospitalar de Lisboa Ocidental, Lisboa - Portugal

**Keywords:** Aortic Valve Stenosis/complications, renal Insufficiency,Chronic, Calcinosis, Renal Dialysis, Diabetes Mellitus, Cardyomyopathies, Hypertension

## Abstract

**Background:**

Chronic kidney disease (CKD) is frequently present in patients with aortic valve disease. Decreased kidney perfusion as a consequence of reduced cardiac output may contribute to renal dysfunction in this setting.

**Objective:**

Given the potential reversibility of kidney hypoperfusion after valve repair, this study aimed to analyze the impact of percutaneous transcatheter aortic valve implantation (TAVI) on kidney function.

**Methods:**

We performed a retrospective analysis of 233 consecutive patients who underwent TAVI in a single center between November 2008 and May 2016. We assessed three groups according to their baseline estimated glomerular filtration rate (eGFR) (mL/min/1.73 m^2^): Group 1 with eGFR ≥ 60; Group 2 with 30 ≤ eGFR < 60; and Group 3 with eGFR < 30. We analyzed the eGFR one month and one year after TAVI in these three groups, using the Chronic Kidney Disease Epidemiology Collaboration (CKD-EPI) formula to calculate it.

**Results:**

Patients from Group 1 had a progressive decline in eGFR one year after the TAVI procedure (p < 0.001 vs. pre-TAVI). In Group 2 patients, the mean eGFR increased one month after TAVI and continued to grow after one year (p = 0.001 vs. pre-TAVI). The same occurred in Group 3, with the mean eGFR increasing from 24.4 ± 5.1 mL/min/1.73 m^2^ before TAVI to 38.4 ± 18.8 mL/min/1.73 m^2^ one year after TAVI (p = 0.012).

**Conclusions:**

For patients with moderate-to-severe CKD, kidney function improved one year after the TAVI procedure. This outcome is probably due to better kidney perfusion post-procedure. We believe that when evaluating patients that might need TAVI, this ‘reversibility of CKD effect’ should be considered.

## Introduction

Since Bright^1^ first described the association between chronic kidney disease (CKD) and heart disease in 1836, many epidemiological studies have confirmed and extended this finding.

With higher life expectancy, the prevalence of valvular heart disease, such as aortic valve disease, is increasing, and patients needing intervention are older and display multiple comorbidities.^2^ Surgical intervention is the most effective therapeutic option, but transcatheter aortic valve implantation (TAVI) has become an important treatment choice for inoperable or high-risk patients.^2-4^

Many studies show poor short- and long-term outcomes in patients with CKD submitted to TAVI.^5,6^ Other studies on this field focus on acute kidney injury (AKI) after TAVI, showing that AKI is not merely an independent predictor of adverse outcome but also predisposes to the development of CKD. Cases of AKI requiring dialysis have a poor prognosis (50% in-hospital mortality), and a significant proportion of patients progress to end-stage kidney disease.^7-9^

Aortic valve disease is frequently seen in CKD patients^10^ due to progressive and accelerated leaflet calcification, a well-known complication of kidney failure. The key modulators in this field have not been totally identified, but might include calcification inhibitors (e.g., fetuin-A and matrix Gla protein), calcification promotors (e.g., hyperphosphatemia, calcium-phosphate product, parathyroid hormone), and leptin. On the other hand, long-standing aortic stenosis may contribute to CKD by impairing forward blood flow from the heart, causing chronic hypoperfusion and resulting in organ damage, and by increased renal venous pressure associated with right-sided heart failure.^11,12^ Hypothetically, these pathological CKD mechanisms can be reversed after correction of aortic valve stenosis.

Little is known about the reversibility of CKD after aortic valve replacement. The dynamic changes in kidney function after TAVI have not been described and are not fully understood.

Given the potential reversibility of the pathological CKD mechanism after the correction of aortic valve disease, this study aimed at analyzing the variations in kidney function after TAVI.

## Methods

**Figure 1:**
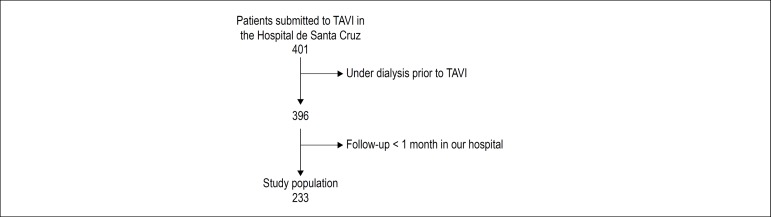
Flowchart of the patient population. TAVI: transcatheter aortic valve implantation.

We performed a retrospective analysis of patients submitted to TAVI at the Hospital de Santa Cruz - Centro Hospitalar de Lisboa Ocidental, Lisbon, Portugal, between November 2008 and May 2016. We excluded patients under dialysis prior to the procedure and those with a follow-up of less than one month in our center (Figure 1).

Demographic and clinical data were collected from patient chart review. All patients met standard indications for aortic valve replacement.

TAVI was performed mainly by a transfemoral approach. Transapical, subclavian, and transaortic accesses were used in case the former approach was not adequate due to calcification, tortuosity, or caliper. Delivery catheters between 14 F and 20 F sizes were used for valve delivery after previous aortic valve stenosis crossing with a guidewire. Preparation by valvuloplasty with an undersized aortic valve balloon was left to the discretion of the operators, as well as post-dilation valvuloplasty. Several types of valves were selected according to anatomic, valvular, and clinical characteristics based on computed tomography angiography and/or transesophageal echocardiogram (TEE): self-expandable, balloon, and mechanically expandable devices were implanted (respectively Corevalve^®^/Corevalve Evolut^®^/Portico^®^, Edwards^®^, and Lotus^®^) in the cath lab by a team including an experienced interventional cardiologist and cardiac surgeons, under fluoroscopic guidance and discretionary intraprocedural TEE. The protocol of the center determined the type (Iomeron® or Visipaque®) and volume (mL) of the iodine contrast selected.

Patient baseline characteristics included demographic data and comorbidities, such as diabetes, coronary artery disease, peripheral vascular disease, hypertension, chronic heart failure, and obesity (Body Mass Index ≥ 30 kg/m^2^). Comorbidities found in patient charts were classified in accordance with the International Classification of Diseases, Ninth Revision (ICD-9). Kidney function was assessed by estimated glomerular filtration rate (eGFR), which was calculated with the Chronic Kidney Disease Epidemiology Collaboration (CKD-EPI) formula^13^ using the closest serum creatinine (sCreat) within 5 days prior to the procedure and after 1 and 12 months (1 year). Based on pre-TAVI eGFR, we evaluated three groups according to the categories suggested by the Kidney Disease: Improving Global Outcomes (KDIGO) 2012 guidelines:^13^ Group 1 with eGFR ≥ 60 mL/min/1.73 m^2^ (patients without CKD or CKD G1-2); Group 2 with 30 ≤ eGFR < 60 mL/min/1.73 m^2^ (CKD G3a-b); and Group 3 with eGFR<30 (CKD G4-5). Start of renal replacement therapy (RRT) and mortality during follow-up were also considered.

Categorical variables were expressed as frequency distributions and percentages, and continuous variables as mean ± standard deviation. Continuous variables as median values were tested using the paired Student’s t-test, and categorical variables were compared with the chi-square test. Differences in eGFR among the three groups over time were analyzed using repeated measures ANOVA. Sphericity was determined by the Mauchly's test when the p-value > 0.05. When the Mauchly’s test did not identify sphericity, we used repeated measures ANOVA with Greenhouse-Geisser correction. Multivariate logistic regression was generated for analyses predictors of eGFR improvement.

All statistical tests used the software SPSS version 22.0 (IBM Corp., Armonk, NY, USA). We considered p < 0.05 statistically significant.

## Results

We analyzed data from 233 consecutive patients submitted to TAVI in a single center in Lisbon, Portugal, from November 2008 to May 2016.

Table 1 summarizes the baseline characteristics of the patients. The mean age of the patients was 81.8 ± 7.5 years (47 to 94 years), and 56.7% were females. Among all patients, 30.5% had diabetes; 40.3%, coronary artery disease; 22.3%, peripheral vascular disease; 69.5%, hypertension; 35.2%, chronic heart failure; and 17.2% were obese. The mean sCreat was 1.2 ± 0.49 mg/dL, and the mean eGFR was 55.2 ± 19.9 mL/min/1.73 m^2^. During the follow-up period, 26.6% of patients died.

**Table 1:** Baseline characteristics

	All patients (n = 233)	Group 1 (n = 100)	Group 2 (n = 101)	Group 3 (n = 32)	P-value
Females, n (%)	132 (56.7)	49 (49)	66 (64.7)	17 (54.8)	0.078
Age (years, mean ± SD)	81.8 ± 7.5	80.0 ± 9.2	83.5 ± 5.6	81.7 ± 4.9	0.003
Diabetes, n (%)	71 (30.5)	27 (27)	31 (30.4)	13 (41.9)	0.30
Coronary artery disease, n (%)	94 (40.3)	36 (36)	45 (44.1)	13 (41.9)	0.46
Peripheral vascular disease, n (%)	52 (22.3)	18 (18)	28 (27.5)	6 (19.4)	0.23
Hypertension, n (%)	162 (69.5)	73 (73)	70 (68.6)	19 (61.3)	0.46
Chronic heart disease, n (%)	82 (35.2)	30 (30)	37 (36.7)	15 (48.4)	0.16
Obesity, n (%)	29 (17.2)	14 (14)	14 (13.7)	1 (5.9)	0.43
sCreat	1.2 ± 0.49	0.85 ± 0.16	1.26 ± 0.26	2.13 ± 0.45	< 0.001
eGFR	55.2 ± 19.9	74.6 ± 9.5	45.3 ± 8.4	25.0 ± 4.5	< 0.001
Iodine contrast volume (mL)	144.8 ± 82.8	152.7 ± 101.2	139.9 ± 65.1	134.5 ± 64.7	0.434
Dead n (%)	62 (26.6)	29 (29)	21 (20.6)	12 (38.7)	0.11

sCreat: serum creatinine; eGFR: estimated glomerular filtration rate.

Before the TAVI procedure, 100 patients were in Group 1, 101 in Group 2, and 32 in Group 3. The three groups did not present differences regarding gender, incidence of comorbidities, and mortality (Table 1).

Mean eGFR in Group 1, Group 2, and Group 3 before TAVI was 74.6 ± 9.5 mL/min/1.73 m^2^, 45.3 ± 8.4 mL/min/1.73 m^2^, and 25.0 ± 4.5 mL/min/1.73 m^2^, respectively (p < 0.001).

The mean volume of iodine contrast was 144.8 ± 82.8 mL, with no differences in the three groups (p = 0.434). Out of all patients, 54.5% received Iomeron®, and 45.5% received Visipaque®. In Group 1, 65.0% of patients received Iomeron®, and 35.0% received Visipaque® (p = 0.004). In Group 2 and Group 3 patients, there was no difference between the iodine contrast used (p = 0.092 and p = 0.151, respectively) (Table 2).

**Table 2:** Iodine contrast administered to the three groups

	Iomeron® (n; %)	Visipaque® (n; %)	p-value
Group 1	65; 65.0%	35;35.0%	0.004
Group 2	42; 41.2%	62; 58.8%	0.092
Group 3	20; 64.5%	11; 35.5%	0.151

The TAVI procedure had a significant effect on kidney function in the three groups. Sphericity was assumed by Mauchly’s test in Group 1 and Group 3 [c^2^ (2) = 4.34, p = 0.144, c^2^ (2) = 0.54, p = 0.763[. Greenhouse-Geisser correction was used in Group 3 [c^2^ (2)=6.93, p = 0.031].

**Figure 2:**
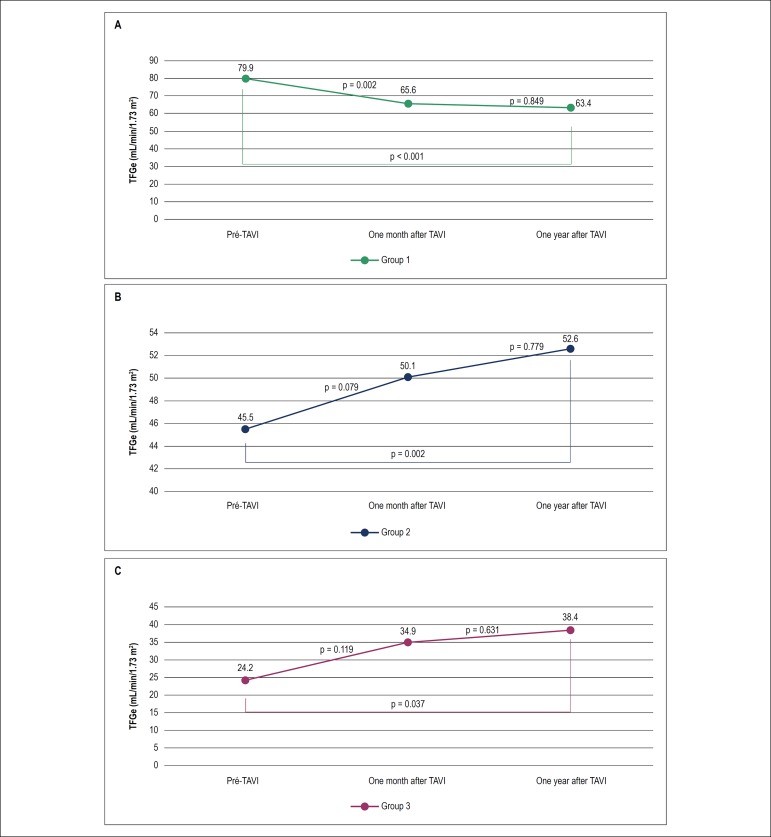
Comparison of eGFR between groups after the TAVI procedure. A: Group 1; B: Group 2; C: Group 3. eGFR: estimated glomerular filtration rate; TAVI: transcatheter aortic valve implantation.

Patients from Group 1 showed a progressive decrease in eGFR after TAVI [F (2-118) = 12.77, p < 0.001], reaching a value of 63.4 ± 19.2 mL/min/1.73 m^2^ one year after the procedure (Table 3 and Table 4). The decline in kidney function was more significant in the first month after the TAVI procedure (Table 4 and Figure 2-A).

**Table 3:** Evolution of kidney function after TAVI

	N patients	eGFR pre-TAVI (mL/min/1.73 m^2^)	eGFR 1 month after TAVI (mL/min/1.73 m^2^)	eGFR 1 year after TAVI (mL/min/1.73 m^2^)	p-value
Group 1	60	74.9 ± 9.0	65.6 ± 20.0	63.4 ± 19.2	<0.001
Group 2	48	45.4 ± 8.5	50.1 ± 15.1	52.6 ± 16.4	0.001
Group 3	17	24.4 ± 5.1	34.9 ± 18.1	38.4 ± 18.8	0.012
All patients	125	56.7 ± 20.5	55.5 ± 20.9	55.8 ± 19.9	0.51

eGFR: estimated glomerular filtration rate; TAVI: transcatheter aortic valve implantation.

*p-value between eGFR pre-TAVI and eGFR 1 year after TAVI.

**Table 4:** Repeated Measures ANOVA: pairwise comparisons (Group 1)

(I) eGFR	(J) eGFR	Mean Difference (I-J)	Std. Error	Sig.^†^	95% Confidence Interval for Difference^†^	
					Lower Bound	Upper Bound
eGFR pre-TAVI	eGFR 1 month after TAVI	9.276*	2.533	0.002	3.034	15.518
	eGFR 1 year after TAVI	11.521*	2.612	< 0.001	5.084	17.958
eGFR 1 month after TAVI	eGFR pre-TAVI	-9.276*	2.533	0.002	-15.518	-3.034
	eGFR 1 year after TAVI	2.245	2.072	0.849	-2.861	7.351
eGFR 1 year after TAVI	eGFR pre-TAVI	-11.521*	2.612	<0.001	-17.95	-5.084
	eGFR 1 month after TAVI	-2.245	2.072	0.849	-7.351	2.861

* The mean difference is significant at the 0.05 level.

† Adjusted for multiple comparisons: Bonferroni. eGFR: estimated glomerular filtration rate; TAVI: transcatheter aortic valve implantation.

Patients from Group 2 presented an increase in eGFR [F (2-94) = 6.25, p = 0.003] one month and one year after TAVI (Table 5). The difference between eGFR means was higher one month after the procedure (Figure 2-B). Group 3 had the same results, that is, the mean eGFR increased over time after the procedure [F (2-32) = 5.91, p = 0.014], and the improvement in kidney function was greater in the first month (Table 6 andFigure 2-C).

**Table 5:** Repeated Measures ANOVA: pairwise comparisons (Group 2)

(I) eGFR	(J) eGFR	Mean Difference (I-J)	Std. Error	Sig.^†^	95% Confidence Interval for Difference^†^	
					Lower Bound	Upper Bound
eGFR pre-TAVI	eGFR 1 month after TAVI	-4.716	2.019	0.079	-9.728	0.295
	eGFR 1 year after TAVI	-7.201*	2.007	0.002	-12.184	-2.219
eGFR 1 month after TAVI	eGFR pre-TAVI	4.716	2.019	0.071	-0.295	9.728
	eGFR 1 year after TAVI	-2.485	2.178	0.779	-7.893	2.923
eGFR 1 year after TAVI	eGFR pre-TAVI	7.201*	2.007	0.002	2.219	12.184
	eGFR 1 month after TAVI	2.485	2.178	0.779	-2.923	7.893

* The mean difference is significant at the 0.05 level.

† Adjusted for multiple comparisons: Bonferroni. eGFR: estimated glomerular filtration rate; TAVI: transcatheter aortic valve implantation.

**Table 6:** Repeated Measures ANOVA: pairwise comparisons (Group 3)

(I) eGFR	(J) eGFR	Mean Difference (I-J)	Std. Error	Sig.^†^	95% Confidence Interval for Difference^†^	
					Lower Bound	Upper Bound
eGFR pre-TAVI	eGFR 1 month after TAVI	-10.453	4.670	0.119	-22.938	2.031
	eGFR 1 year after TAVI	-13.923*	4.944	0.037	-27.138	-0.708
eGFR 1 month after TAVI	eGFR pre-TAVI	10.453	4.670	0.119	-2.031	22.938
	eGFR 1 year after TAVI	-3.470	2.658	0.631	-10.576	3.636
eGFR 1 year after TAVI	eGFR pre-TAVI	13.923*	4.944	0.037	0.708	27.138
	eGFR 1 month after TAVI	3.470	2.658	0.631	-3.636	10.576

* The mean difference is significant at the 0.05 level.

† Adjusted for multiple comparisons: Bonferroni.  eGFR: estimated glomerular filtration rate; TAVI: transcatheter aortic valve implantation.

A multivariate analysis adjusted for gender, age, and comorbidities did not change the variations in eGFR across the three groups.

In a logistic regression model for patients whose kidney function worsened after one month and one year, the contrast administered was a predictor of worsening. Administration of Iomeron**®** was a predictor of worsening in renal function after one year (HR 4.397, 95%CI 1.584-7.286, p = 0.002). On the other hand, the volume administrated was not a predictor of worsening in eGFR after one month (HR 0.997, 95%CI 0.994-1.001, p = 0.125) and one year (HR 0.999, 95%CI 0.995-1.002, p = 0.476).

The incidence of patients needing to initiate dialysis twelve months after the TAVI procedure was 2.4% (five patients). Before TAVI, one of these patients was in Group 1; two were in Group 2; and two were in Group 3. We did not find a statistically significant difference in mortality among the three groups (p=0.11). All of these patients had chronic heart failure, and four died.

## Discussion

This analysis contains data from patients who underwent TAVI in a single center from November 2008 to May 2016. The present results suggest that kidney function might improve in patients with CKD G3-5 after the correction of aortic stenosis. However, in patients with no CKD or with CKD G1-2 (eGFR ≥ 60 mL/min/1.73 m^2^), the eGFR decreased during the follow-up. This study also shows a low incidence of new dialysis - 2.4% (five patients).

Several studies address the prognosis and factors that influence mortality and other poor outcomes in patients with CKD undergoing aortic valve replacement, but little is known about the effect of the treatment of aortic valve disease on kidney function.

This study reveals that our patients with CKD G3-5 (eGFR < 60 mL/min/1.73 m^2^) had an improvement in kidney function one month after aortic valve replacement, maintaining the improvement after one year of follow-up. Other studies have also indicated this potential reversibility of CKD, both early and after one year of follow-up.^2,14-16^

A study with 69 patients from a single center in Brazil^14^ showed an acute kidney recovery after the TAVI procedure. After one year of follow-up, all patients who had an acute recovery remained with improved levels of sCreat. This work also suggests that kidney recovery is more frequent in patients who had more severe renal dysfunction before aortic valve replacement. Azarbal et al.^15^ have found similar results. In their work, acute kidney recovery (defined as a positive change in eGFR of ≥ 25% 48 hours after TAVI) was strongly associated with baseline CKD: 8.9% in patients with eGFR > 60 mL/min/1.73 m^2^ compared to 26.6% in patients with eGFR < 60 mL/min/1.73 m^2^. Also, in a multivariate logistic regression model, lower baseline eGFR was highly predictive of acute kidney recovery (OR 3.27, 95%CI 1.84-5.82, p < 0.001).^15^

Najjar et al.^16^ showed that patients with moderate and severe CKD (30 ≥ eGFR > 60 and eGFR < 30, respectively) had initial improvement in eGFR, peaking one week after the aortic valve replacement. The improvement was maintained after one year for patients with moderate CKD and after six months in patients with severe CKD compared with the pre-TAVI eGFR value. The group with severe CKD also presented a better short- and long-term survival in this study.

We believe that these results are due to an improvement in cardiac output and a reduction in venous congestion after aortic valve replacement, leading to better kidney perfusion, and therefore an improvement in kidney function. These data suggest that a better kidney function can be expected in patients with CKD G3-5, which may have important implications in the selection of individuals for the treatment of aortic valve diseases.

The short- and long-term prognosis of aortic valve replacement in patients with CKD prior to the procedure often calls into question the benefit of valve repair in these patients. Recently, some studies have shown that the poor prognosis associated with CKD is influenced by the stage of the disease.^5,6,15,16^ Gibson and his work group^19^ revealed that eGFR < 60 mL/min/1.73 m^2^ is an important predictor of mortality post-TAVI (HR 5.0, 95%CI 1.87-13.4, p = 0.001) as well as in short-term follow-up (HR 2.98, 95%CI 1.85-4.80, p < 0.001). Other recent study^20^ shows that for patients with eGFR < 60 mL/min/1.73 m^2^, a variation as small as 5 mL/min/1.73 m^2^ in eGFR could make a measurable difference in risk of death, RRT, or both at 30 days and 1 year of follow-up. Nguyen et al.^21^ showed that a worsening in renal function was associated with increased in-hospital mortality, hospital length of stay, and intensive care unit length of stay in surgical aortic valve replacement patients, but not in TAVI patients. Our study contradicts these data. We found no difference in mortality among patients with CKD G3-5 compared to those who had CKD G1-2 or no CKD before TAVI.

Regarding the administration of contrast, the three groups showed no differences regarding the volume received; thus, volume was not a predictor of worsening in eGFR after one month and one year. The predictive value of contrast volume for kidney dysfunction after TAVI is controversial:^15,22,23^ in a meta-analysis with over 3,800 patients post-TAVI, higher contrast use was not clearly associated with a greater risk of AKI.^24^

However, we found a difference in the type of contrast administered in Group 1: most patients with CKD G1-2 at baseline received Iomeron® and this iodine contrast was a predictor of worsening in eGFR. Iodine contrast is divided into three groups according to their osmolarity. Iomeron® is a low-osmolar contrast characterized by values within 300-900 mOsm/kg H_2_O.^25^ Visipaque® is iso-osmolar, having an osmolarity level similar to that of blood (290 mOsm/kg H_2_O) and dimeric structure opposed to monomeric low-osmolar contrast media.^25^ Despite the many years of experience in the use of iodine contrast, the exact pathogenesis of contrast-induced nephropathy (CIN) remains unknown. The causes might include the osmotic effect of contrast media on the kidneys, the increased levels of vasoconstrictive factors, such as adenosine or endothelin, the reduced levels of vasodilators, such as nitric oxide or prostacyclin, and the toxic effect of contrast molecules on renal tubules.^25^ According to the American College of Radiology guidelines, iso-osmolar iodixanol has no evident superiority over low-osmolar contrast with respect to the incidence of CIN.^26^ Regardless, the difference in the contrast administrated may be one of the factors contributing to the poorer results in patients from Group 1, although there are not enough data to prove this supposition, namely whether these patients had AKI after the procedure. Another hypothesis that could explain the kidney function variation in patients with CKD G1-2 is that, prior to the aortic valve repair, they could not tolerate the angiotensin-converting enzyme inhibitor (ACEi) or angiotensin II receptor antagonists (ARA-II), and as such, the therapeutic could be optimized after the procedure, thus explaining the GFR variation.

We found an incidence of new dialyses of 2.4% (five patients) after a year of follow-up in all categories of CKD without statistical difference between them. A recent study in this field showed a difference in new-dialysis patients according to their CKD stage, with an incidence of 1.2%, 3.74%, 14.6%, and 60.1% in CKD 1-2, CKD 3, CKD 4, and CKD 5, respectively.^18^ Given the low incidence of patients who started dialysis in the follow-up period after TAVI, drawing statistically relevant conclusions would not be accurate; nevertheless, we believe that some of these results stand out: (i) the mean age of these patients was 80 ± 5.96 years, similar to the mean age of all the analyzed population (81.8 ± 7.5 years); (ii) almost all patients died (4 out of 5); (iii) all patients had chronic heart failure, which probably contributed to the outcome.

The main limitations of this study concern its retrospective and observational nature. The use of patient charts for data collection is also a limitation, as some data might be missing or incorrectly coded. In addition, a significant number of patients were excluded, which could introduce a systematic bias toward the patients included in the study. Also, sCreat fluctuates often day-to-day, as it is influenced by numerous factors, such as hydration state, medication, or comorbidities. These variations in sCreat significantly affect the estimated kidney function. The present study also has some limitations regarding the patients’ follow-up: short follow-up period (one year); the decline in kidney function with age may be a confounding factor for the true benefit of aortic valve replacement in these patients; and other important covariates not included in this study (such as the severity of aortic stenosis, intra-procedure events, including hypotension, and AKI after the procedure).

Summarizing, the association between worse outcomes in CKD patients undergoing TAVI is well-established, while the potential reversibility of kidney function after aortic valve replacement has not been well-investigated. Despite the limitations, our study provides some significant evidence of reversibility of CKD after aortic valve replacement, probably due to improved renal perfusion post-procedure. Further randomized controlled studies involving more patients and longer follow-up periods are necessary to evaluate the reversibility of CKD after aortic valve replacement.

## Conclusions

Our study suggests that the correction of aortic stenosis is associated with an improvement in kidney function in patients with moderate to severe CKD, showing some significant evidence of reversibility of CKD after aortic valve replacement. The confirmation of this ‘reversibility of CKD’ effect is clinically important insofar as it may help to improve the decision-making process, refining risk stratification in these challenging groups of patients, and perhaps become one of the indications for TAVI.
